# Pregnancy outcomes before and after institution of a specialised twins clinic: a retrospective cohort study

**DOI:** 10.1186/s12884-015-0654-5

**Published:** 2015-09-11

**Authors:** Amanda Henry, Nicole Lees, Kendall J. Bein, Beverley Hall, Veronica Lim, Katie Qiao Chen, Alec W Welsh, Lisa Hui, Antonia W. Shand

**Affiliations:** School of Women’s and Children’s Health, UNSW Medicine, Kensington, NSW Australia; Department of Maternal-Fetal Medicine, Royal Hospital for Women, Sydney, NSW Australia; Department of Emergency Medicine, Royal Prince Alfred Hospital, Sydney, NSW Australia; Midwifery and Women’s Health Nursing Research Unit, University of Sydney, Sydney, Australia; Department of Perinatal Medicine, Mercy Hospital for Women, Heidelberg, VIC Australia; Department of Obstetrics and Gynaecology, University of Melbourne, Melbourne, Australia

## Abstract

**Background:**

Although specialised clinics for multiple pregnancies are recommended by several Obstetrics and Gynaecology governing bodies, studies examining outcome before and after introduction of such clinics remain few, were performed predominantly in North America in the 1990s, and either amongst dichorionic twin pregnancies only or where chorionicity was not specified. Our objective, in the modern setting with twins of known chorionicity, was to compare maternal and neonatal outcomes of twin pregnancies before and after commencement of a consultant-led, multidisciplinary twins clinic (TC).

**Methods:**

Retrospective cohort study of 513 women, with birth of twins at ≥20 weeks’ gestation, January 2007 to November 2011, at a metropolitan tertiary maternity hospital, Sydney, Australia. Demographic, pregnancy, and outcome data were obtained from hospital databases. Women receiving TC care (2009–2011) were compared to those receiving general antenatal clinic (ANC) care (2007–2010) and private care (2009–2011). Other models of care were excluded. Main outcome measures were total maternal inpatient stay, mode of birth, gestational age at birth, and neonatal nursery admission.

**Results:**

286 women were included in the main analyses: 84 attended ANC, 101 TC, and 101 a private obstetrician. TC women had similar demographics to ANC women and were slightly younger than private patients. TC women had lower Caesarean section rates (55 % vs. 70 % ANC and 76 % private, *p* = 0.008) and fewer late preterm (34 + 0–36 + 6 weeks) births, (26 %TC vs. 44 % ANC and 41 % private, *p* < 0.001). Median maternal inpatient stay was shorter in TC than ANC (7 vs. 8 days, *p* = 0.009) and similar to private (7 days). Nursery admission rates were higher in private patients (67 % vs. 49 % ANC and 47 % TC, *p* = 0.001) and average birthweight lower (2283 g vs. 2501 g ANC and 2496 g TC, *p* < 0.001).

**Conclusions:**

Within a single centre, maternal and neonatal twin pregnancy outcomes varied significantly by model of care. Introducing a specialised twins clinic in our setting decreased Caesarean section rates, late preterm birth, and inpatient stay compared to ANC.

**Electronic supplementary material:**

The online version of this article (doi:10.1186/s12884-015-0654-5) contains supplementary material, which is available to authorized users.

## Background

There has been a well-documented increase in the incidence of twin and higher order multiple gestations in recent decades, related primarily to advancing maternal age and use of assisted reproductive technologies [1, 2]. Multiple gestations carry significantly higher risks for both the mother and fetuses [3], leading to recommendations for specialised antenatal care for multiple pregnancies. National Institute for Health and Care Excellence (NICE) guidelines in the United Kingdom recommend that “*clinical care for women with twin and triplet pregnancies should be provided by a nominated multidisciplinary team consisting of [practitioners with] experience and knowledge of managing*” such pregnancies [4].

However, as acknowledged by the guideline authors, the evidence base for this recommendation is sparse. A 2012 Cochrane review identified only one small randomised trial, which did not show significant benefit of midwifery-led education and specialised care on perinatal outcomes, and which found an increased Caesarean rate in the intervention group [5]. Several non-randomised studies suggest some benefit to specialised antenatal care for multiple pregnancies, including reduction in very low birth weight [6], extreme prematurity [7], perinatal mortality [6], neonatal intensive care admission [6, 7] and neonatal costs [7, 8].

None of the retrospective studies include data collected in the past 10 years, during which time early determination of chorionicity and differential surveillance and management of monochorionic pregnancies has become standard care [4, 9]. The profile of confounding factors such as maternal age, body mass index (BMI) and comorbidities has also changed markedly in this time. Further examination of twin clinic outcomes is therefore essential.

In March 2009, a dedicated maternal-fetal medicine specialist led, multidisciplinary Twins Clinic (TC) commenced at the Royal Hospital for Women (RHW), Sydney, Australia.

The current study’s objective was to compare pregnancy outcomes of women with either dichorionic or monochorionic twin pregnancy, stratified by model of care: antenatal clinic (ANC), twins clinic (TC) and private care. We hypothesised that consistent specialised care through a dedicated twins clinic would reduce length of any antenatal admissions and decrease postpartum stay. We also wished to examine whether TC care would impact on major perinatal outcomes including mode of birth, gestation at birth, neonatal nursery admission, and perinatal mortality.

## Methods

A retrospective cohort study was performed at RHW, a metropolitan tertiary hospital in Sydney, Australia that delivers approximately 4000 women per annum. It has a neonatal intensive care unit and a maternal-fetal medicine department.

The twins clinic is led by a specialist in maternal-fetal medicine and provides standardised multi-disciplinary antenatal care for women with a twin pregnancy, based upon the 2008 RCOG monochorionic pregnancy guideline and the 2006 RCOG consensus workshop [10, 11]. Regular ultrasound assessment for fetal growth and wellbeing are performed in the clinic (every 2 weeks for monochorionic twins and every 4 weeks for dichorionic twins). A checklist ensures specific points of information are provided during the course of the pregnancy, including a detailed discussion of delivery plans undertaken in the third trimester. All women are encouraged to attend specific multiple pregnancy antenatal classes. Mode and timing of delivery plans are clearly documented in patient records, with elective delivery (either induction of labour or planned Caesarean) for uncomplicated pregnancies being offered at 37–38 weeks for dichorionic twins and 36–37 weeks for monochorionic twins.

Prior to the establishment of the twins clinic, women with twin pregnancies were cared for in the hospital’s general antenatal clinic, by a number of obstetricians. Women with twins having private care also delivered at the hospital throughout the study period. In both these models of care, antenatal care was at the discretion of the individual obstetrician.

The study included all women with a twin pregnancy who delivered ≥20 weeks’ gestation at RHW from 1^st^ Jan 2007 to 1^st^ November 2011.

**Fig. 1:**
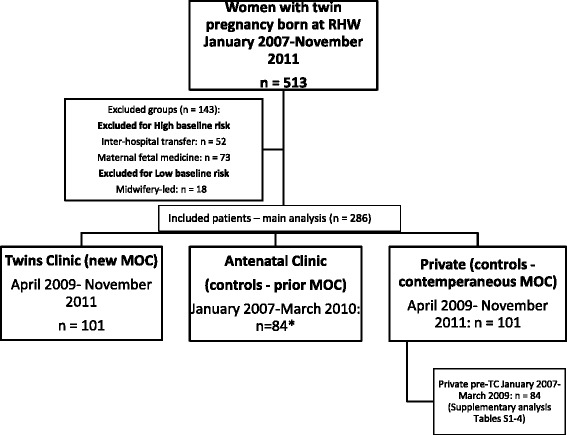
Flowchart of women in the study

Women were classified by models of antenatal care. Obstetrician-led care included ANC, TC, private care, maternal-fetal medicine (MFM), and inpatient interhospital transfers. Uncomplicated DCDA twins were also eligible for midwifery-led care during the study period. Women initially triaged to MFM care (e.g. because of early-onset growth restriction or twin-twin transfusion syndrome), inpatient interhospital transfers (antenatal care at another hospital) and midwifery-led care were excluded from the study, as they had different baseline levels of risk to TC/ANC. A flow chart (Fig. 1) outlines the study inclusions and exclusions. ANC women were offered transfer to TC in March-April 2009 when TC commenced, and TC was the default model of care for new twin antenatal bookings from April 2009. Eleven women remained within ANC during this transition period at their and/or ANC consultant request. Analysis of demographics and primary outcome with and without including these 11 women did not alter the demographic results, so the decision was made to include all 84 ANC women to allow for greater statistical power for study outcomes.

Maternal demographic data and pregnancy outcomes were obtained from the hospital’s obstetric database ObstetriX (ObstetriX consortium, NSW Health), with missing information obtained from other electronic databases (eMR, Cerner Systems) or patient records. The ObstetriX database contains information about pregnancies of greater than or equal to 20 weeks’ gestation, or 400 grams birthweight where the gestational age is not known. Data is entered by the midwife at time of booking for antenatal care, at birth and upon discharge from hospital.

As the major aim of establishing TC was to provide consistent, evidence-based care of twin pregnancy, it was expected that TC care would decrease inpatient admissions and length of stay. The primary outcome was therefore total length of maternal inpatient admission throughout the pregnancy (antenatal admissions and birth admission). Secondary outcomes were (a) mode of birth (b) gestational age at birth and (c) neonatal nursery (special care or neonatal intensive care) admission. Other pregnancy outcomes analysed included fetal anomaly, perinatal death (stillbirth or death prior to neonatal discharge), maternal pregnancy hypertension (pre-eclampsia and gestational hypertension), gestational diabetes, and birth by Caesarean section. A stillbirth was defined as a birth ≥20 weeks’ gestation with no signs of life at birth, or ≥400 g when the gestational age was not known. Maternal hypertension and preeclampsia were defined according to the 2008 Society of Obstetric Medicine of Australia and New Zealand classification [12].

The major comparisons are between (a) TC (April 2009 to November 2011), (b) the predominantly historical control of ANC (January 2007 to March 2010: small proportion of women continued with ANC after TC establishment), and (c) contemporaneous control of private women (April 2009 to November 2011). Comparisons were performed both among all 3 groups of the cohort, and between ANC and TC alone, and TC vs. private alone. Supplementary comparisons were also performed between historical (January 2007 to March 2009) private patients and contemporaneous private patients (April 2009 to November 2011) to examine whether outcomes observed appeared merely related to epoch/time-related changes in care, or were likely related to the care given.

Statistical analysis was performed using Excel and SPSS version 21 (SPSS Inc., Chicago, IL). Significant differences between categorical variables were tested using Chi-squared tests, or the Fisher exact test. Significant differences between continuous variables were tested using Student’s *t*-test or ANOVA for normally distributed data, and using Kruskal-Wallis test for non-parametric data. All tests were two-sided, statistical significance was defined as probability value of <0.05 with 95 % confidence intervals (95 % CI).

Local institutional review board (ethics committee) approval (South Eastern Sydney Local Health District Human Research Ethics Committee Reference Number 09/177) was obtained, including waiver of requirement for participant consent.

## Results

Figure 1 shows the study flowchart. Of the 513 women (1026 babies) delivered at RHW at ≥20 weeks gestation during the study period, 286 were in the three major included groups: ANC (84 women), TC (101 women), and post-TC Private (101 women). Of the remaining 227 women, 143 women were excluded from further analysis (125 high-risk transfers in or MFM, 18 low-risk midwifery-led care of DCDA twins), and 84 were pre-TC private patients compared in supplementary analyses to post-TC private patients (Additional file 1: Tables S1–S4).

Demographic characteristics by model of care and year of birth are shown in Table 1. There were no significant differences in demographic characteristics between women attending ANC and TC. Mean maternal age and the proportion of mothers born in Australia were higher in the contemporaneous control group (private patients April 2009 to November 2011) than in the TC and ANC groups.

**Table 1:** Demographic characteristics for the women by model of care and year of birth

	Model of Care			
	ANC	TC	PRIVATE	*p* value^a^
	(2007–2010)	(2009–2011)	(2009–2011)	
Baseline characteristics *n* = 286	*N* = 84	*N* = 101	*N* = 101	
Maternal age at delivery, years (mean ± SD)	32.5 ± 4.7	32.6 ± 4.5	35.8 ± 4.5	<0.001
BMI pre-pregnancy (kg/m^2^) (mean ± SD)	24.8 ± 6.7	24.3 ± 5.1	23.6 ± 3.2	0.29
	N (%)	N (%)	N (%)	
Born in Australia	44 (52)	57 (56)	73 (72)	<0.001
Nulliparous	52 (62)	61 (60)	65 (64)	0.84
Chorionicity				
DCDA	66 (79)	70 (69)	71 (72)	0.32
MCDA	16 (19)	31 (31)	28 (28)	0.18
MCMA	0 (0)	0 (0)	0 (0)	1
Unknown	2 (2)	0 (0)	2 (2)	

*SD* standard deviation, *BMI* body mass index, *DCDA* dichorionic, diamniotic, *MCDA* monochorionic, diamniotic, *MCMA* monochorionic, monoamniotic

^a^Overall ANC Vs. TC Vs. Private comparison (*ANC* antenatal clinic, *TC* twins clinic)

Maternal outcomes are presented in Table 2. There were no significant differences between frequency of maternal pregnancy complications, or admission with an antenatal complication, between TC and ANC. However, Caesarean section rates were significantly lower in the TC cohort compared to other cohorts (55 % TC vs. 70 % ANC and 78 % private, *p* = 0.008). There were higher rates of labour (versus delivery without preceding labour) in the ANC and TC groups (48 and 54 %) compared to private (35 %, *p* = 0.02), and correspondingly higher rates of induction or augmentation of labour. Steroid administration was significantly higher in the private cohort (47 % vs. 23 % TC, *p* = 0.002) despite fewer reported episodes of threatened premature labour. There were no decreases in other complications or admission rates in the private group.

**Table 2:** Maternal outcomes by model of care and year of birth

	Model of care			
	ANC	TC	PRIVATE	
	(2007–2010)	(2009–2011)	(2009–2011)	
	*N* = 84	*N* = 101	*N* = 101	*p* value^a^
Maternal outcomes	Number (%)	Number (%)	Number (%)	
Antenatal admission	46 (55)	60 (59)	49 (49)	0.3
Complications of pregnancy				
Placenta praevia	3 (4)	0 (0)	2 (2)	0.18
Antepartum haemorrhage ≥ 20 weeks	6 (7)	3 (3)	3 (3)	0.28
Hypertensive disorder of pregnancy	11 (13)	13 (13)	13 (13)	1
Gestational diabetes	7 (8)	9 (9)	5 (5)	0.51
Threatened premature labour	18 (21)	14 (14)	6 (6)	0.008
PROM	11 (13)	13 (13)	8 (8)	0.43
Antenatal steroids	27 (32)	23 (23)	47 (47)	0.002
Labour	40 (48)	54 (54)	35 (35)	0.02
Labour induced or augmented	27 (35)	44 (44)	24 (24)	0.01
Mode of birth				
Caesarean both twins	56 (67)	54 (54)	74 (73)	0.01
Vaginal birth both twins^b^	25 (30)	45 (45)	25 (25)	0.008
Caesarean second twin after vaginal birth Twin 1	3 (4)	2 (2)	2 (2)	0.73
Any Caesarean	59 (70)	56 (55)	76 (76)	0.008
Epidural use in labour	23 (56)	37 (69)	26 (74)	
	(Total *N* = 40)	(Total *N* = 54)	(Total *N* = 35)	0.29
PPH 500–999 ml	21 (25)	28 (28)	20 (20)	0.41
PPH ≥ 1000 ml	5 (6)	10 (10)	11 (11)	0.74
Maternal length of stay				
Total admission length (days), median (IQR)	8.0 (6–12)	7 (6–9)	7 (5–9)	0.001
Total admission length ≥ 7 days, n (%)	58 (69)	52 (52)	64 (63)	0.04
Maternal postnatal stay (days), median (IQR)	5.8 (4.5–7.0)	5.1 (4.6–6.1)	5.7 (4.9–6.8)	0.004
Maternal postnatal stay ≥ 5 days, n (%)	55 (65)	60 (59)	70 (69)	0.33

*PROM* premature rupture of membranes, *PPH* Postpartum haemorrhage, *IQR* Interquartile range

^a^Overall ANC Vs. TC Vs. private comparison (*ANC* antenatal clinic, *TC* twins clinic)

^b^ includes normal vaginal birth, instrumental, vaginal breech

Total inpatient stay for the pregnancy (antenatal, intrapartum and postnatal) was lower in TC than ANC patients, median 7 days (IQR 5–9) vs. 8 days (IQR 6–12), *p* = 0.009. TC women were also less likely to have a total stay of 7 days or more than either ANC or Private (52 % TC, 69 % ANC, 63 % Private, *p* = 0.04), and had a slightly shorter mean postnatal stay (5.3 ± 1.7 days TC vs. 6.0 ± 2.0 days ANC and 5.8 ± 1.6 days Private, *p* = 0.001).

Fetal and neonatal outcomes are shown in Table 3. Perinatal mortality was low, and similar between groups. Median gestation at birth was similar for ANC vs. TC, and lower in private patients (36.6 weeks ANC vs. 37.1 weeks TC and 36.0 weeks private, *p* = 0.003). Rates of late prematurity (34 + 0–36 + 6 weeks gestation) were significantly lower in TC women compared to both ANC and Private cohorts (TC 26 %, ANC 44 %, Private 41 %, *p* < 0.001).

**Table 3:** Fetal and neonatal outcomes by model of care and year of birth

	ANC	TC	PRIVATE	*p* value^a^
	2007–2010	2009–2011	2009–11	
	*n* = 168	*n* = 202	*n* = 202	
Fetal Outcomes	N (%)	N (%)	N (%)	
Fetal anomaly	0 (0)	2 (1)	6 (3)	0.04
Gestation at birth (weeks), median (IQR)	36.6 (35.3–37.5)	37.1 (35.3–37.6)	36.0 (34.1–37.1)	0.003
Gestation categories:				
<28 weeks	0 (0)	4 (2)	0 (0)	0.03
28–33 + 6 weeks	16 (10)	28 (14)	46 (24)	0.002
34–36 + 6 weeks	74 (44)	52 (26)	83 (41)	<0.001
37+ weeks	78 (46)	118 (58)	73 (36)	<0.001
Gender				
Male	85 (51)	103 (51)	99 (49)	0.92
Birthweight mean ± SD (g)	2501 ± 499	2486 ± 586	2283 ± 547	<0.001
Birthweight categories				
0–999 g	1 (1)	5 (3)	3 (2)	0.35
1000–1499 g	4 (2)	9 (5)	13 (6)	0.18
1500–2499 g	69 (41)	80 (40)	105 (52)	0.03
≥2500 g	94 (56)	108 (54)	81 (41)	0.004
Apgar <5 at 5 min	0 (0)	2 (1)	3 (1)	0.31
Nursery admission at birth	82 (49)	95 (47)	129 (64)	0.001
Stillbirth	0 (0)	0 (0)	3 (2)	0.06
Neonatal death	0 (0)	2 (1)	0 (0)	0.16
Perinatal mortality	0 (0)	2 (1)	3 (2)	0.3
Breastfeeding at discharge	144 (86)	173 (86)	185 (92)	0.12

*IQR* Interquartile range, *SD* Standard deviation

^a^Overall ANC Vs. TC Vs. Private comparison (*ANC* antenatal clinic, *TC* twins clinic)

Birthweight and nursery admission rates were also similar for ANC and TC, however birthweight was approximately 200 g lower in private patients reflecting the earlier gestation at birth, and nursery admission rates higher (49 % ANC, 47 % TC, 64 % Private, *p* = 0.001).

All comparisons were also made between historical (pre-TC) private patients and contemporaneous private patients (supplementary data). Average maternal age was slightly higher in the later private cohort (35.8 vs. 33.8 years, *p* = 0.03), however other demographic characteristics were similar. There were no clinically significant differences in the major maternal and neonatal outcomes to suggest epoch/time-related changes in care during the study period.

## Discussion

### Main findings

Our study demonstrates that multidisciplinary care in a specialised twins clinic was associated with significant differences in maternal and neonatal outcomes compared to conventional models of care. Maternal outcome differences included reduction in Caesarean section rate and reduced total maternal inpatient stay. Regarding fetal outcomes, reduction in late prematurity was significant. These differences were achieved without any apparent increase in maternal or fetal complications.

### Maternal outcomes

Although Caesarean rates were higher in both ANC and private vs. TC, the reasons appeared to differ. Given similar rates of labour and induction/augmentation in ANC vs. TC, increased ANC intrapartum Caesarean rates likely accounted for the difference. Regarding TC and private women, more TC women underwent labour, with the difference between these cohorts probably due to increased private elective Caesarean rates. We cannot exclude factors such as a higher rate of non-vertex first twins in private women (with consequent Caesarean section), however it is unlikely this would account for the entire observed 19 % difference. Importantly, many of these Private elective Caesareans were occurring late preterm (34 + 0–36 + 6 weeks), with the late preterm and term Caesarean rates being 82 and 63 % respectively for Private women (Vs. 42 and 54 % respectively in TC women). The increased late preterm elective (predominantly Caesarean) birth in turn partially accounts for increased neonatal nursery admission rates in the Private cohort. However, 28 + 0–33 + 6 week preterm birth was also more common in the Private cohort (and at that gestation Caesarean rate was approximately 80 % in all groups). Although it is not possible from this study to obtain reasons for the high rate of late preterm elective Caesareans in the Private cohort, it may relate to lower tolerance of risk, particularly as regards MCDA twins.

This study was undertaken prior to publication of the Twin Birth trial [13], and TC clinicians discussed with women the available epidemiological evidence about perinatal outcomes and mode of birth [14, 15]. These standardised discussions and clear documentation of delivery plan may have increased confidence of TC women and staff regarding intrapartum care, resulting in decreased intrapartum Caesarean rate compared to ANC. Certainly substantially more vaginal deliveries for TC women involved a vaginal breech birth (11 % vs. 1 % ANC, *p* < 0.001), possibly reflecting greater acceptance of second twin vaginal breech birth in this group.

Although modest on an individual basis, the one day reduction of total pregnancy maternal inpatient stay in TC compared to ANC has considerable health system economic implications. The average Australian cost of a pregnancy/childbirth admission in 2011 (length of stay 2.5 days) was AUD 4815 (GBP 3100.86, USD 4978.00, EUR 3572.99 on average 2011 interbank rate) [16], so a consistently shorter stay represents substantial savings. Clearly it is not ascertainable from the study databases the reason for the length of stay reduction, although it was found both slightly shorter antepartum admissions and slightly shorter postpartum stay contributed. We speculate that the specialised TC care led to greater confidence amongst women and clinicians regarding appropriate earlier discharge for both antenatal admissions and the birth admission. This is supported by the smaller interquartile range for length of stay in TC compared to ANC.

### Fetal outcomes

We found a significant reduction in late preterm birth in TC. As high risk twin pregnancies were triaged to MFM care, and there were no differences in antenatal complications, the reason for this difference in prematurity is unclear. It may be due to greater confidence in methods of fetal surveillance while awaiting term delivery in the TC cohort. Given emerging concerns regarding consequences of late prematurity, we speculate that TC care may confer important long term benefits [17, 18].

### Strengths and limitations

Study strengths include the reasonably large cohort, known chorionicity derived in high proportion from ultrasound reports and placental pathology, and use of the private patient groups to help overcome any historical cohort effect.

The limitations of this study include its retrospective and non-randomised nature, as differences in outcome might be secondary to differences in patient characteristics, and/or confounders such as general improvement in outcomes over time. Regarding underlying characteristics, there were no significant TC and ANC demographic differences, and although private women were slightly older, maternal complications such as hypertension did not differ between groups. Additionally, chorionicity did not differ between TC and private, making it less likely that the outcome differences noted can be explained by different risk profiles between groups.

Regarding time-related versus care-related findings, analysis was performed both of private controls being seen in the TC era, and of private women being seen January 2007 to March 2009 (pre-TC) versus April 2009 to November 2011. Demographic characteristics of pre-TC and TC private women were similar apart from the later cohort being older. No substantive differences in private women’s maternal or fetal outcomes were noted over time. This makes it less likely that the change in outcomes that occurred after TC introduction reflects only a general time-related improvement in outcomes.

As model of care was not randomised, selection bias is also a potential limitation. Although all women booked with ANC were offered transfer to TC when it commenced, and TC booking was the default model of care for public bookings after March 2009, 11 of 84 ANC women had their babies after TC established (of whom 7 were already beyond 20 weeks’ gestation when TC commenced). As retrospective note review does not reveal reasons for women staying with ANC, selection bias is possible: however, demographic factors and chorionicity did not differ between these women and the total ANC cohort, and the initial 2009 TC women (*n* = 15) were if anything of higher risk profile than subsequent TC women (53 % Vs. 27 % MCDA, *p* = 0.07), so unlikely to bias findings in favour of TC. The decision was therefore made to include all ANC and TC women in the outcome analysis, without excluding the crossover time period.

The choice of which models of care to include could also be questioned. This was based on an attempt to compare populations of broadly equivalent risk i.e. ANC, TC and Private. As women triaged to MFM <20 weeks would usually be because of a likely fetal complication, and women transferred in from another hospital would usually be secondary to complications such as premature labour, these populations were considered inherently high-risk and not an appropriate comparison. Conversely, a small proportion of low-risk women were looked after in midwifery-led care (permitted for DCDA twins if the woman had booked into this model of care, wished to remain in this model of care, and had no major maternal conditions or initial known obstetric complications), so were considered of lower baseline risk and not appropriate for direct comparison. As there were only 18 such women (6 pre-TC, 12 in TC era) it is unlikely their exclusion has substantially altered the overall study results: however, is a further reason why a randomised trial would be the preferred method of demonstrating utility of a multiple pregnancy clinic Vs. standard care.

As for all retrospective studies, both accuracy and scope of database-derived data may be a limitation. As fields such as chorionicity, model of care, perinatal mortality, and maternal inpatient stay were checked against multiple sources, and fields relating to labour and birth have been shown to be accurate in prior local research [19], we are confident of the data pertaining to our primary and major secondary outcomes. However, some data appear contradictory: in particular, the rate of threatened premature labour (TPL) was lower in Private patients than TC or ANC while other markers of threatened or actual preterm birth (e.g. steroid administration, gestation at birth) are higher. As the “threatened premature labour” field may encompass both admitted and non-admitted cases, has yes/no/unsure input rather than more detailed information, and might be more specifically asked about/known for public rather than private patients by the midwives filling in the database, we believe this result likely reflects the limitations of the database rather than a true difference in TPL rates between groups.

Further limitations include the lack of formal patient satisfaction information (although TC women did report favourably on the convenience of having ultrasound and visit scheduled at the same time), or formal economic analysis. Both of these would ideally be addressed using a prospective, randomised design in a centre where equipoise regarding TC care still exists.

### Interpretation in light of other evidence

Prior comparable studies are summarized in Table 4 [6–8, 20]. In the one published RCT (162 women) of specialised antenatal care for multiple pregnancy, the additional midwifery care and information trialled did not improve maternal or fetal morbidities [20]. However, this trial was underpowered for such outcomes and was not a trial of specialised obstetric antenatal care. Other studies regarding the value of specialist multiple pregnancy care are, like this study, retrospective in nature, and all performed in the USA [21]. Outcomes vary, but all are broadly supportive of improvement in fetal and/or maternal outcomes, although none found decreased Caesarean rate. However, all also either included only DCDA twins, or had no chorionicity information available. Given the importance of chorionicity in determining twin pregnancy outcome [9], lack of this information makes interpretation of these studies especially problematic.

**Table 4:** Prior studies of specialised twin pregnancy care

	Sen et al., 2005 [20]	Ellings et al., 1993 [6]	Ruiz et al., 2001 [7]	Luke et al., 2003 [8]
Study design	RCT	Retrospective cohort – Twins Clinic and contemporaneous ANC patients	Retrospective historical cohort – Twins clinic and ANC pre Twins clinic	Retrospective cohort – multiple pregnancy clinic vs. contemporaneous ANC patients
Number of women	80 specialised care, 82 standard care	89 TC, 51 ANC	30 TC, 41 ANC	190 TC, 339 ANC
Chorionicity data	Not available	Not available	Not available	Only DCDA twins included
Interventions studied	Midwifery-led antenatal and postnatal visits, patient education	Multidisciplinary MFM-led care, consistent protocols including dietary, evaluation of maternal symptoms and cervical status, patient education	Nurse practitioner care, standard protocols, weekly visits from 24 weeks, home visit for social assessment	Fortnightly visits, dietary supplementation and advice, patient education
Significant Findings	Increased Caesarean Section rate	Decreased perinatal mortality (1 vs. 8 %), decreased incidence birthweight <1500 g (6 vs. 26 %), decreased NICU admission (13 vs. 38 %)	Decreased premature birth <30 weeks (0 vs. 29 %) and <36 weeks (32 vs. 41 %), decreased neonatal length of stay and cost	Multiple improved maternal and fetal outcomes including decreased preeclampsia, higher birthweight, lower serious neonatal morbidity rates, decreased cost/twin of care, less rehospitalisation or developmental delay to age 3
	No significant change in other maternal or fetal outcomes			
		No difference maternal antenatal complications		

Although the value of decreased length of stay, decreased rates of prematurity, and decreased nursery admission rates found in this study are widely accepted, whether a decrease in Caesarean rates for twin pregnancy is desirable has been contested, given epidemiological data regarding increased perinatal morbidity and mortality for the second-born twin [22, 23]. However, Barrett et al’s recent large RCT found no significant difference in fetal, neonatal or maternal outcomes between elective Caesarean and planned vaginal delivery for twins ≥32 weeks where Twin 1 was vertex presentation [13]. Caesarean section has recognized implications for subsequent pregnancies and is generally more costly to the healthcare system than vaginal birth [24]. Therefore our observed reduction in Caesarean deliveries, without an increase in adverse events, should indeed be viewed as a positive outcome.

Although our study supports the establishment of specialised Twins Clinics, further research is required. An economic analysis of the establishment of a Twins Clinic, examining whether reduced inpatient stay, prematurity, and Caesarean rates offset clinic implementation costs (including staff education and ultrasound service provision), is essential for long-term viability of this model of care. While one prior study found a substantial decrease in immediate neonatal costs after specialised antenatal care for twin pregnancy was introduced, neither maternal cost of care nor implementation costs were assessed [7], and we are not aware of any prior studies where a full economic evaluation of a specialised multiple pregnancy clinic has been performed. Ultimately, a sufficiently powered, randomised controlled trial of Twins Clinic versus other models of care will be needed to confirm the benefits identified in studies such as ours.

## Conclusions

This study supports the use of specialised clinics for the antenatal care of women with twin pregnancies. It suggests that a dedicated multidisciplinary twins clinic can lead to reductions in total maternal inpatient length of stay, late prematurity, and Caesarean section rates, without an increase in maternal or neonatal complications.

## Additional file

Additional file 1:
**Private patients prior to Twins Clinic (January 2007 to March 2009) and during Twins Clinic era (April 2009 to Dec 2011).** (DOCX 21 kb)

## Abbreviations

TC: Twins Clinic
ANC: Antenatal Clinic
NICE: National Institute for Health and Care Excellence
BMI: Body Mass Index
RHW: Royal Hospital for Women
RCOG: Royal College of Obstetricians and Gynaecologists
MFM: Maternal-fetal medicine
DCDA: Dichorionic, diamniotic
MCDA: Monochorionic, diamniotic
MCMA: Monochorionic, monoamniotic
AUD: Australian Dollar
GBP: British Pound
USD: United States Dollar
RCT: Randomised Controlled Trial
USA: United States of America

## References

[1] Kulkarni A, Jamieson D, Jones H (2013). Fertility treatments and multiple births in the United States. N Engl J Med.

[2] Glinianaia SV, Rankin J, Sturgiss SN (2013). The north of England survey of twin and multiple pregnancy. Twin Res Hum Genet.

[3] Modena AB, Berghella V (2005). Antepartum management of multifetal pregnancies. Clin Perinatol.

[4] National Collaborating Centre for Women’s and Children’s Health (2011). Multiple pregnancy: the management of twin and triplet pregnancies in the antenatal period.

[5] Dodd JM, Crowther CA. Specialised antenatal clinics for women with a multiple pregnancy for improving maternal and infant outcomes. Cochrane Database Syst Rev. 2012;8. 10.1002/14651858.CD005300.pub3 22895946

[6] Ellings J, Newman R, Hulsey T, Bivins H, Keenan A (1993). Reduction in very low birth weight deliveries and perinatal mortality in a specialized, multidisciplinary twin clinic. Obstet Gynecol.

[7] Ruiz R, Brown C, Peters M, Johnston A (2001). Specialized care for twin gestations: improving outcome and reducing costs. J Obstet Gynecol Neonatal Nurs.

[8] Luke B, Brown MB, Misiunas R (2003). Specialized prenatal care and maternal and infant outcomes in twin pregnancy. Am J Obstet Gynecol.

[9] Moise KJ, Johnson A (2010). There is NO diagnosis of twins. Am J Obstet Gynecol.

[10] RCOG (2008). Management of Monochorionic twin pregnancy.

[11] Consensus views arising from the 50th Study Group: multiple pregnancy. In: Kilby M, Baker P, Critchley H, Field D, editors. Multiple pregnancy. London: RCOG Press; 2006. p. 283–6.

[12] Lowe SA, Brown MA, Dekker GA (2009). Guidelines for the management of hypertensive disorders of pregnancy 2008. Aust N Z J Obstet Gynaecol.

[13] Barrett JF, Hannah ME, Hutton EK (2013). A randomized trial of planned cesarean or vaginal delivery for twin pregnancy. N Eng J Med.

[14] Smith G, Pell JP, Dobbie R (2002). Birth order, gestational age, and risk of delivery related perinatal death in twins: retrospective cohort study. BMJ.

[15] Smith G, Shah I, White IR, Pell JP, Dobbie R (2005). Mode of delivery and the risk of delivery‐related perinatal death among twins at term: a retrospective cohort study of 8073 births. BJOG.

[16] Independent Hospital Pricing Authority. National hospital cost data collection Australian public hospitals cost report 2011–2012, Round 16. Canberra, Australia: Commonwealth of Australia; 2014.

[17] Chan E, Quigley MA. School performance at age 7 years in late preterm and early term birth: a cohort study. Archives of Disease in Childhood-Fetal and Neonatal Edition 2014: fetalneonatal-2014-306124. 10.1136/archdischild-2014-306124 24966128

[18] Vohr B (2013). Long-term outcomes of moderately preterm, late preterm, and early term infants. Clin Perinatol.

[19] Roberts CL, Bell JC, Ford JB, Morris JM (2009). Monitoring the quality of maternity care: how well are labour and delivery events reported in population health data?. Paediatr Perinat Epidemiol.

[20] Carrick‐Sen D, Steen N, Robson S (2014). Twin parenthood: the midwife’s role–a randomised controlled trial. BJOG.

[21] Bricker L (2014). Optimal antenatal care for twin and triplet pregnancy: the evidence base. Best Pract Res Clin Obstet Gynaecol.

[22] Smith G, Fleming KM, White IR (2007). Birth order of twins and risk of perinatal death related to delivery in England, Northern Ireland, and Wales, 1994–2003: retrospective cohort study. BMJ.

[23] Roberts CL, Algert CS, Nippita TA, Bowen JR, Shand AW (2015). Association of prelabor cesarean delivery with reduced mortality in twins born near term. Obstet Gynecol.

[24] Souza JP, Gülmezoglu A, Lumbiganon P (2010). Caesarean section without medical indications is associated with an increased risk of adverse short-term maternal outcomes: the 2004–2008 WHO Global Survey on Maternal and Perinatal Health. BMC Med.

