# Supplementary material for: Pregnancy outcomes before and after institution of a specialised twins clinic: a retrospective cohort study
Source: BMC Pregnancy Childbirth. 2015 Sep 11;15:217. doi: 10.1186/s12884-015-0654-5 (PMC4567816; doi:10.1186/s12884-015-0654-5)
Supplement: Additional file 1: — Private patients prior to Twins Clinic (January 2007 to March 2009) and during Twins Clinic era (April 2009 to Dec 2011). (DOCX 21 kb) [file 12884_2015_654_MOESM1_ESM.docx]

**Additional file 1: Private patients prior to Twins Clinic (January 2007-March 2009) and during Twins Clinic era (April 2009-Dec 2011)**

**Table S1: Demographics Private pre-TC vs post-TC**

| **Baseline Characteristic** |  |  | **Model of Care** | |
| --- | --- | --- | --- | --- |
|  | **Private pre-TC** | | **Private post-TC** | **P value** |
| **Mean Maternal age at delivery (±SD)** | 33.8 ± 4.5 | | 35.8 ± 4.5 | **0.003** |
| **BMI pre-pregnancy (mean±SD)** | 24.0 ± 4.8 | | 23.6 ± 3.2 | 0.51 |
|  | *n (%) total n = 84* | | *n (%) total n = 101* |  |
| **Born in Australia** | 51 (61) | | 73 (72) | 0.10 |
| **Nulliparous** | 32 (38) | | 36 (36) | 0.73 |
| **Chorionicity** | | | | |
| DCDA | 61 (72) | | 71 (72) | 0.10 |
| MCDA | 13 (15) | | 28 (28) |  |
| MCMA | 3 (4) | | 0 (0) |  |
| Unknown | 7 (8) | | 2 (2) |  |

**Table S2: Maternal outcomes Private pre-TC vs post-TC**

| **Maternal Outcomes** |  |  | **Model of Care** | |
| --- | --- | --- | --- | --- |
|  | **Private pre-TC** | | **Private post-TC** | **P value** |
|  | Number (%)  Total n = 84 | | Number (%)  Total n =101 |  |
| **Antenatal admission** | 49 (57) | | 49 (49) | 0.21 |
| **Complications of pregnancy** |  | | | |
| Placenta praevia | 1 (1) | | 2 (2) | 0.92 |
| APH > 20weeks | 4 (5) | | 3 (3) | 0.70 |
| Hypertensive disorder of pregnancy | 11 (13) | | 13 (13) | 0.96 |
| Gestational diabetes | 8 (10) | | 5 (5) | 0.23 |
| Threatened premature labour | 18 (21) | | 6 (6) | **0.002** |
| PROM | 19 (23) | | 8 (8) | **0.005** |
| Antenatal steroids | 38 (45) | | 47 (47) | 0.86 |
| Cervical shortening | 2 (2) | | 5 (5) | 0.46 |
| **Labour occurred** | 25 (30) | | 35 (35) | 0.48 |
| **Mode of birth (baby)** | **168 babies** | | **202 babies** |  |
| Normal Vaginal | 19 (11) | | 19 (9) | 0.55  0.11  0.57  0.86 |
| Instrumental | 11 (7) | | 23 (11) |  |
| Caesarean | 129 (77) | | 150 (74) |  |
| Vaginal Breech | 9 (5) | | 10 (5) |  |
| **Mode of birth (mother)** | **N = 84** | | **N = 101** |  |
| Caesarean only | 63 (75) | | 74 (73) | 0.79 |
| Normal vaginal birth only | 7 (8) | | 6 (6) | 0.53 |
| Vaginal birth including instrumental or vaginal breech | 11 (13) | | 19 (19) | 0.29 |
| Caesarean for second twin after vaginal birth Twin 1 | 3 (4) | | 2 (2) | 0.51 |
| **Any Caesarean** | 66 (79) | | 76 (75) | 0.43 |
| **Epidural use** | 67 (80) | | 92 (91) | **0.027** |
| **Blood loss** |  | | | 0.23 |
| <500ml | 69 (82) | | 70 (69) |  |
| 500-999ml | 9 (11) | | 20 (20) |  |
| 1000-1499ml | 2 (2) | | 5 (5) |  |
| >1500ml | 4 (5) | | 6 (6) |  |

**Table S3: Fetal outcomes Private pre-TC vs. private post-TC**

| **Fetal Outcomes** |  |  | **Model of Care** | |
| --- | --- | --- | --- | --- |
|  | **Private pre-TC** | | **Private post-TC** | **P value** |
|  | Number (%) Total n = 168 | | Number (%) Total n = 202 |  |
| **Fetal anomaly** | 4 (2) | | 6 (3) | 0.73 |
| **Median Gestation at birth (IQR)** | 35.6 (34.3-37.1) | | 36.0 (34.1-37.1) | 0.23 |
| **Gestation:** |  | | | |
| <28 weeks | 6 (4) | | 0 (0) | **0.02**  0.38  0.09  0.12 |
| 28-33+6 weeks | 32 (19) | | 46 (24) |  |
| 34-36+6 weeks | 82 (50) | | 83 (41) |  |
| 37+ weeks | 48 (29) | | 73 (36) |  |
| **Gender** |  | | | |
| Male | 84 (50) | | 99 (49) | 0.85 |
| Female | 84 (50) | | 103 (51) |  |
| **Birthweight mean±SD** | 2225±614g | | 2283±547g | 0.34 |
| **Birthweight** | p = 0.18 for overall comparison | | | |
| 0-999g | 9 (5) | | 3 (2) | **0.04**  0.79  0.94  0.33 |
| 1000-1499g | 12 (7) | | 13 (6) |  |
| 1500-2499g | 88 (52) | | 105 (52) |  |
| 2500g or more | 59 (35) | | 81 (41) |  |
| **Nursery admission at birth** | 114 (68) | | 129 (64) | 0.42 |
| **Perinatal mortality** | 4 (2) | | 3 (2) | 0.70 |
| **Breastfeeding at discharge** | 144 (86) | | 185 (92) | 0.07 |

**Table S4: Maternal Admission outcomes Private pre-TC vs. private post-TC**

| **Admission Outcomes** |  |  | **Model of Care** | | |
| --- | --- | --- | --- | --- | --- |
|  | **Private pre-TC (n =84)** | | **Private post-TC (n=101)** | | **P value** |
|  | Number | Percent | Number | Percent |  |
| **Median Total Admission length (days)/IQR** | **Median**  7.00 | **IQR**  6.0-10.0 | **Median**  7.00 | **IQR**  6.0-9.0 | 0.609 |
| **Total admission length** |  |  |  |  |  |
| <7 | 33 | 40.2% | 37 | 37.0% | 0.654 |
| 7+ days | 49 | 59.8% | 63 | 63.0% |  |
| **Maternal Postnatal stay (days) median/IQR** | **Median**  5.800 | **IQR**  4.9-6.8 | **Median**  5.700 | **IQR**  5.0-7.0 | 0.952 |
| **Maternal postnatal stay** |  |  |  |  |  |
| <5 days | 11 | 14.9% | 13 | 15.7% | 0.890 |
| 5+ days | 63 | 85.1% | 70 | 84.3% |  |
